# Hotspot Regions of Quantitative Trait Loci and Candidate Genes for Ear-Related Traits in Maize: A Literature Review

**DOI:** 10.3390/genes15010015

**Published:** 2023-12-21

**Authors:** Xingjie Zhang, Jiachen Sun, Yudong Zhang, Jinfeng Li, Meichen Liu, Linzhuo Li, Shaoxiong Li, Tingzhao Wang, Ranjan Kumar Shaw, Fuyan Jiang, Xingming Fan

**Affiliations:** 1 School of Agriculture, Yunnan University, Kunming 650500, China; xingjiezhang2022@163.com (X.Z.); jinfengli1020@163.com (J.L.); shirleyliu1028@163.com (M.L.); lilinzhuo0606@163.com (L.L.); 15987701739@163.com (S.L.); 2 College of Agronomy and Biotechnology, Yunnan Agricultural University, Kunming 650201, China; jiachens5046@163.com (J.S.); wangtz202311@163.com (T.W.); 3 Institute of Food Crops, Yunnan Academy of Agricultural Sciences, Kunming 650205, China; mikezhangy@yahoo.com (Y.Z.); ranjanshaw@gmail.com (R.K.S.); jiangfuyansxx@126.com (F.J.)

**Keywords:** maize, quantitative trait loci, ear-related traits, consistent QTL, candidate gene, hotspot region

## Abstract

In this study, hotspot regions, QTL clusters, and candidate genes for eight ear-related traits of maize (ear length, ear diameter, kernel row number, kernel number per row, kernel length, kernel width, kernel thickness, and 100-kernel weight) were summarized and analyzed over the past three decades. This review aims to (1) comprehensively summarize and analyze previous studies on QTLs associated with these eight ear-related traits and identify hotspot bin regions located on maize chromosomes and key candidate genes associated with the ear-related traits and (2) compile major and stable QTLs and QTL clusters from various mapping populations and mapping methods and techniques providing valuable insights for fine mapping, gene cloning, and breeding for high-yield and high-quality maize. Previous research has demonstrated that QTLs for ear-related traits are distributed across all ten chromosomes in maize, and the phenotypic variation explained by a single QTL ranged from 0.40% to 36.76%. In total, 23 QTL hotspot bins for ear-related traits were identified across all ten chromosomes. The most prominent hotspot region is bin 4.08 on chromosome 4 with 15 QTLs related to eight ear-related traits. Additionally, this study identified 48 candidate genes associated with ear-related traits. Out of these, five have been cloned and validated, while twenty-eight candidate genes located in the QTL hotspots were defined by this study. This review offers a deeper understanding of the advancements in QTL mapping and the identification of key candidates associated with eight ear-related traits. These insights will undoubtedly assist maize breeders in formulating strategies to develop higher-yield maize varieties, contributing to global food security.

## 1. Introduction

Maize (*Zea mays* L.) stands as one of the most widely cultivated crops worldwide. Originating in South America, it has since spread globally [1,2]. The increasing human demand for maize, recognized as a crucial source of food, feed, and industrial raw materials, underscores the importance of increasing yields as a primary objective in maize breeding [3,4]. Advancements in genome sequencing technologies have paralleled the investigation of the localization of quantitative trait loci (QTL) in maize to the forefront of research. Ear-related traits are complex quantitative traits controlled by multiple genes. These traits include ear length (EL), ear diameter (ED), kernel row number (KRN), kernel number per row (KNPR), kernel length (KL), kernel width (KW), kernel thickness (KT), and hundred kernel weight (HKW). Collectively, these factors play a significant role in influencing both the yield and quality of maize [5,6,7]. Research findings indicate that QTLs associated with these traits are clustered on different chromosomes [8]. The rate of phenotypic variation explained (PVE) by these QTLs varies across different traits. Genetic effects are primarily observed as additive effects (A) and dominant (D) effects. The mode of gene action includes the A, D, and partially dominant (PD) effect and the over dominant (OD) effect.

Three QTL mapping methods, namely interval mapping (IM), composite interval mapping (CIM), and inclusive composite interval mapping (ICIM), are commonly employed in studies focusing on maize ear-related QTL studies. IM, introduced by Lander et al. (1989) [9], boasts low population requirements and yields more accurate mapping results in the presence of a solitary QTL. However, in cases where multiple QTLs are in close proximity, interference between them can compromise mapping accuracy. CIM, proposed by Zeng (1994), integrates multiple regression and interval mapping techniques [10]. This method enhances the precision and efficiency of QTL mapping by leveraging marker information across the entire genome while retaining the advantages of interval mapping. However, CIM is limited in that it can only assess one interval at a time, and it lacks the capability to compute interactions between QTLs or between QTLs and environmental factors. ICIM was proposed by Li et al. (2007) [11] and built on the foundation of ICM, strategically positioning the genetic linkage map into multiple intervals for separate analysis. This approach proves effective in mitigating environmental interference, addressing the complexities associated with multiple QTLs, and ultimately achieving more precise mapping results.

Genome-wide association analysis (GWAS) and bulked segregant analysis (BSA) are commonly used methods for rapid mapping of QTLs. GWAS involves a comprehensive examination of common genetic variations across the entire genome of the target individual. This is achieved by analyzing the linkage disequilibrium decay distance between numerous SNP molecular markers throughout the genome and the target site [12]. QTL mapping often struggles to identify minor QTLs, with limitations in resolution, speed, and allele frequency [13]. In contrast, GWAS represents a significant advantage in association mapping. Leveraging historical recombination events accumulated over hundreds of generations, GWAS offers superior resolution and a higher allele frequency [14]. BSA stands out as an economical and swift method for screening linkage markers or mapping QTLs in individuals exhibiting extreme phenotypes [15]. BSA not only significantly reduces workload and costs but also enhances the efficiency of linkage marker screening. It proves to be more economical and rapid compared to both genetic mapping and GWAS. This approach has found widespread application in the QTL mapping and gene mapping of maize. QTL-seq, a cutting-edge technology rooted in next-generation sequencing, integrates the strengths of both BSA and whole genome sequencing. This method allows for the rapid and efficient localization of QTLs [16].

Gene cloning stands as a pivotal technology in molecular biology. In vitro recombination techniques, specific genes, and other DNA sequences are sequentially inserted into the vector, offering a robust tool for investigating gene function and regulatory mechanisms. The prevalent gene cloning methods in plants can be broadly categorized into three groups: gene cloning based on mutant materials, gene cloning grounded in association analysis, and map-based cloning using parental mating linkage groups [17]. Map-based cloning is a strategic approach employed to isolate a specific target gene by progressively narrowing down the mapping interval of the gene or QTL on the chromosome. What sets this method apart is its self-sufficiency, as it does not rely on prior knowledge of the target gene sequence and its expression product information. Consequently, map-based cloning plays a pivotal role in bridging the gap for cloning numerous unknown genes responsible for essential agronomic traits in plants. In the realm of maize ear-related traits QTL gene cloning approach, map-based cloning primarily encompasses two techniques: chromosome walking and chromosome landing [18]. This methodology makes a substantial contribution to deepening our comprehension of the genetic factors that impact maize yield. Moreover, it plays a crucial role in improving maize yield identifying and leveraging key genes [19,20,21]. The evolution of polymerase chain reaction technology and CRISPR-Cas9 technology has further accelerated and refined the process of cloning and verifying target genes, enabling a faster and more precise approach.

Fine mapping, a technique within the realm of map-based cloning, has garnered increasing attention. Numerous studies emphasize the significance of fine mapping for QTLs located in hotspots, stable QTLs, major QTLs, and QTL clusters. Ning et al. (2021) identified a QTL (qEL7) associated with EL. Through the fine mapping of this QTL, the researchers revealed a candidate gene linked to EL, exhibiting a negative regulatory effect on this trait [22]. Chen et al. (2022) found a QTL (qKRN2) within the RIL population of MT-6 and B73. Utilizing the ICIM method, they confirmed that the deletion of the *Zm00001d002641* gene, encoding WD40 protein within this QTL region, resulted in an increase in the ear row number of maize. This genetic modification correspondingly led to a roughly 10%, increase in maize yield, with minimal impact on other traits [23]. Fei et al. (2022) conducted mapping and analysis of plant-type QTLs in maize. Their findings revealed three consistent QTLs associated with plant type, and within these QTLs, three candidate genes linked to plant type were identified. Simultaneously, the study identified QTLs related to both plant height and ear height within the hotspot region bin 3.05 [24]. He et al. (2023) used some stable QTLs and QTL clusters to identify candidate genes affecting water and nitrogen uptake in maize within the context of a QTL mapping study on water and nitrogen interaction [25]. Similarly, Dong et al., in their investigation into the genetic structure and molecular mechanisms underlying maize ear traits, discovered 16 candidate genes within 17 QTL clusters. These findings hold significant value for enhancing maize yield [26].

A large number of QTLs related to maize ear traits have been identified by previous researchers. Despite this, the successful cloning of QTLs remains relatively limited. Consequently, the identification and cloning of key genes controlling the variation in ear traits are crucial. This process not only aids in unraveling the molecular mechanisms underpinning maize yield formation but also facilitates the exploration of excellent alleles. Ultimately, these efforts provide theoretical guidance and essential genetic resources for the practice of high-yield breeding in maize [27,28]. Fine mapping is a technique within the framework of map-based cloning. Map-based cloning is a strategy employed to clone a specific target gene by continuously narrowing down the mapping interval of the gene or QTL on the chromosome. What distinguishes this method is its independence from prior knowledge of the target gene sequence and its expression product information. As such, map-based cloning serves as a crucial bridge for cloning the majority of unknown genes that govern essential agronomic traits in plants. It stands out as one of the most widely used gene cloning technologies [18]. Fine mapping and candidate gene cloning, implemented through a map-based cloning strategy, offer an accelerated approach to dissecting the genetic foundation of maize yield genes. These methodologies contribute significantly to advancing our understanding of the genetic factors influencing maize yield. 

The objective of this study was to comprehensively review and analyze hotspot regions, candidate genes, major and stable QTLs, and QTL clusters, focusing on the advancements in QTL mapping for eight ear-related traits in maize over the past 30 years. The results of this statistical analysis can assist subsequent researchers in gaining a deeper understanding of the genetic mechanisms underlying QTLs for ear traits in maize. Additionally, it facilitates the expedited identification of major QTLs, thereby hastening progress toward the goal of breeding maize varieties with high and stable yields. Furthermore, this study identifies current challenges in the research of ear-related traits and proposes possible solutions, offering valuable insights and theoretical references for further investigations into QTLs associated with these traits.

## 2. Materials and Methods

The statistical data presented in this paper mainly refer to the maize B73 RefGen_ v3 genome. For each trait, the QTL bins were enumerated from the relevant literature using Excel. In cases where QTL bins were not specified in the literature, their corresponding bin regions were identified using molecular markers or physical distances in the Bin Viewer toolbar of maize GDB (http://www.maizegdb.org/, accessed from 20 August 2023 to 15 October 2023). Bin regions appearing in at least five distinct literatures were classified as hotspots. All bin positions’ QTLs were compiled in an Excel table for statistical analysis. Subsequently, the R Studio software’s RIdeogram package was employed to generate a QTL distribution map related to ear-related traits based on hot bins. In cases where candidate genes from the literature lacked a specified physical location, the maize GDB unified search was utilized to assign the corresponding physical location, employing the unit “bp” for uniformity.

## 3. QTLs for Eight Ear-Related Traits in Maize

The aforementioned studies reveal the identification of one to twenty-one QTLs associated with EL across different populations. Predominantly, these QTLs are situated on 10 chromosomes and 10 with chromosomes 1, 2, 3, and 10 exhibiting a higher frequency of QTLs related to EL compared to other chromosomes. Individual QTLs for Ed exhibited a PVE ranging from 2.10% to 31.50%, as determined by ED-related QTL studies. Chromosomes 1, 2, 3, 4, 8, 9, and 10 were identified as the primary locations for QTLs related to ED, with single QTLs explaining phenotypic variation in ED from 4.40% to 24.00%. The genetic effects of these QTLs for ED were predominantly additive in nature. The QTL studies for KRN commonly employed ICIM as the mapping method. The distribution of QTLs associated with KRN spanned across all chromosomes, with a notable concentration on chromosomes 2, 4, 5, 8, and 9. The PVE of individual QTLs ranged from 1.40% to 36.76%. The QTL mapping analysis for KRN often utilized SSR molecular markers and the ICIM method. In the case of KNPR, fewer QTLs were distributed on chromosomes 2, 4, and 6, with a predominant presence on chromosomes 1 and 7. A single QTL for KNPR could elucidate 0.40% to 29.65% of the PVE. The QTLs associated with four kernel traits, KL, KW, KT, and HKW, have been discussed earlier. QTLs for KL were mainly distributed on chromosomes 1, 2, 3, and 9, with a single QTL explaining 0.46% to 21.67% of the phenotypic variation. QTLs for KW were primarily distributed on chromosomes 1, 3, 4, and 5, and a single QTL could account for a PVE ranging from 0.81% to 23.69%. Among the four kernel traits, KT exhibited the highest number of identified QTLs, mainly on chromosomes 1, 2, 4, and 8, and only one QTL was found on chromosome 6. The PVE for a single QTL in KT ranged from 0.84% to 22.92%. For HKW, QTLs were mainly distributed on chromosomes 1, 2, and 7, with a single QTL explaining a PVE ranging from 0.46% to 21.45%.

### 3.1. QTLs for Ear Length

Austin and Lee (1996) used the F_6:7_ families derived from Mo17 and H99 to construct a mapping population. Using the IM method and restriction fragment length polymorphism (RFLP) markers, six QTLs related to EL were detected on chromosomes 1, 2, 4, 6, and 8, and the phenotypic variation explained (PVE) by these QTLs for EL ranged from 2.10% to 5.60% [29]. Yang et al. (2005) utilized the F_2:3_ family lines of maize derived from 48-2 and 5003 as a mapping population for QTL mapping using the IM method and detected four QTLs related to EL on chromosomes 1, 2, 4, and 10, with the PVE ranging from 11.00 to 31.50% for individual QTLs. They also observed intergenic roles for A, PD, and OD, with PD being the dominant one [30]. Ren et al. (2015) employed a recombination inbred line (RIL) constructed from maize 178 and 9782 to locate QTLs for maize ear traits under different phosphorus levels. Two QTLs for EL were located on chromosomes 4 and 8 under normal phosphorus, explaining 6.04% to 7.53% of the phenotypic variation. Under low phosphorous conditions, two QTLs for EL were located on chromosomes 6 and 8, explaining 5.44–8.76% of the phenotypic variation [31]. Yi et al. (2019) used the RIL and IF_2_ (Immortalized F_2_, IF_2_) populations of maize derived from 08-641 and Ye478 to conduct a single-environment mapping analysis of maize yield QTLs. Fifteen QTLs related to EL were mapped in the RIL population with the PVE of these QTLs ranging from 3.19 to 11.29%. A total of 21 QTLs for EL were detected in the IF_2_ population, explaining 2.67–13.64% of the phenotypic variation for EL. Five QTLs were co-located in both the RIL and IF_2_ populations [32]. Mei et al. (2021) used the ICIM method for QTL analysis in the F_2_ and F_2:3_ populations of maize derived from the cross between Yi16 and B73. Two QTLs associated with ear length were detected in the F_2_ population, with PVE values of 8.40% and 9.30%, and one QTL associated with ear length was detected in the F_2:3_ population, with a PVE of 9.70% [33]. Sa et al. (2021) conducted QTL mapping in a RIL population developed by crossing Mo17 and KW7 using the ICIM method and detected a QTL related to ear length on chromosome 6, with a PVE value of 18.87% [34].

### 3.2. QTLs for Ear Diameter

Veldboom and Lee (1994) used RFLP markers to construct a genetic map utilizing F_2:3_ families derived from Mo17 and H99. Six QTLs related to ear diameter were mapped by the IM method. These QTLs were distributed on chromosomes 1, 2, 3, 6, 7, and 8, explaining 10.00% to 24.00% of the PVE for ED [35]. Li et al. (2007) used the F_2_ and BC_2_F_2_ populations derived from Dan232 and N04 and used SSR markers for QTL mapping using the CIM method. Six QTLs related to ED were detected in the BC_2_F_2_ population, which explained 4.40% to 16.0% of the PVE for ED. Four QTLs related to ED were detected in the F_2_ population, which explained 7.10–14.80% of the PVE for ED [36]. Zhang et al. (2010) used the F_9_ RIL population constructed by crossing Mo17 and Huangzaosi to map QTLs related to ED under two nitrogen environments by the ICM method. Under normal nitrogen conditions, three QTLs for ED were mapped, contributing 5.68–8.70% to the ED phenotype. Under nitrogen stress, two QTLs for ear diameter were detected, explaining 6.08% to 6.89% of the PVE for ED. One QTL for ED was located on chromosome 9 under both nitrogen environments [37]. Mendes-Moreira et al. (2015) used the F_2:3_ families derived from PB260 and PB266 to locate QTLs in two environments using the IM method, utilizing both SSR and RFLP markers. They found four QTLs for ED in both environments, with a PVE ranging from 8.70% to 19.10% [38]. Su et al. (2017) used an F_2_ population derived from SG-5 and SG-7, employing SNP markers for QTL mapping. QTL mapping for eight maize yield-related traits based on the CIM method identified five QTLs for ED, with a PVE ranging from 6.40% to 11.60% [39]. Following the approach of Su et al. (2017), Zhao et al. (2019) employed identical parental lines, mapping methods, and molecular markers to identify and map four QTLs associated with ED in the F_2:3_ population, and the PVE of these QTLs ranged from 8.50% to 12.00% [40]. Jiang et al. (2023) utilized the F_7_ RIL population of maize constructed by crossing Y32 and Ye107, and three ED QTLs were located using the CIM method, and the PVE values ranged between 7.10% and 10.02% [41].

### 3.3. QTLs for Kernel Row Number

Beavis et al. (1994) employed the IM method to identify QTLs in F_2:3_ families derived from B73 and Mo17 and located four QTLs for KRN on chromosomes 4, 5, 7, and 9, explaining 8.00–10.00% of the phenotypic variance [42]. Yan et al. (2006) used an F_2:3_ population of maize derived from Zong3 and 87-1 to locate yield QTLs using multiple interval mapping (MIM), and identified eight QTLs for KRN, with a PVE ranging from 5.80 to 13.20% [43]. Karen et al. (2008) used the compositive interval mapping in F_2:3_ families derived from L-08-05F and L-14-4B and identified 10 QTLs for KRN, with a PVE ranging from 2.40 to 16.90% [44]. Yang et al. (2015) used an F_2_ population of maize derived from B73 and SICAU1212 using the CIM method and detected seven QTLs related to the number of ear rows in two environments, explaining 6.78% to 36.76% of the phenotypic variance [45]. In their study, Chen et al. (2016) [46] established a four-way cross-mapping population using hybrids of D276 and D72, as well as hybrids of A188 and Jiao51. Through this approach, they successfully mapped seven QTLs associated with the number of KRN, explaining phenotypic contribution rates ranging from 4.47% to 11.24% [23]. Zhang et al. (2017) performed QTL localization analysis using an RIL population of maize developed by crossing Ye478 and Qi319 in four environments and detected 10 QTLs related to the number of rows in ears, explaining 5.39–7.79% of the phenotypic variation [47]. Nie et al. (2019) used NIL-1133B and B73 to map QTLs for ear traits in maize. They successfully identified one QTL for KRN in both F_2_ and F_2:3_ populations [48]. Han et al. (2020) constructed an F_2:3_ population by crossing V54 and Lian87 and mapped twenty-two kernel row number QTLs in four environments, with a PVE range of 1.40% to 14.95% [49]. Zhao et al. (2021) used F_2:3_ families of maize derived from T32 and Qi 319 and identified four QTLs for KRN, with a PVE ranging from 4.13 to 8.33% [50].

### 3.4. QTLs for Kernel Number per Row

Yan et al. (2006) detected eight QTLs for KNPR in two environments, explaining 5.40% to 11.80% of the phenotypic variation, using F_2:3_ families derived from Zong3 and 87-1 [51]. Dai et al. (2009) used the CIM method to locate QTLs in an F_2_ population constructed from maize L26 and 095 inbred lines, and a total of three QTLs for KNPR were detected on chromosomes 1, 9, and 10, explaining 17.01% to 29.65% of the phenotypic variation [52]. Wang et al. (2015) utilized two F_2:3_ populations derived from TY6 and Mo17 (TM population) and TY6 and W138 (TW population) for the QTL mapping of maize yield-related traits by the CIM method. Three QTLs were detected in the TM population and seven QTLs were detected in the TW population, with the PVE of KNPR ranging from 0.40% to 17.70%, including four QTLs with PVE values greater than 10% [53]. In their study, Huo et al. (2016) conducted QTL mapping analysis using Mo17 and W138 in the F_2:3_ population established by TY16. They identified three QTLs for KNPR in the Mo17 and TY16 populations, with a PVE ranging from 3.40% to 17.60%. Additionally, six QTLs for KNPR were detected in the W138 and TY16 populations, explaining 0.40% to 17.80% of the PVE [3]. Zhang et al. (2017) used the IM method to locate five QTLs for KNPR in a single environment to analyze the F_2:3_ families constructed by crossing Baicibaogu and Qiranhuang, which contributed 5.45% to 11.80% of the phenotypic variance for KNPR. The additive effects of the QTLs, all of which were supplied by the parent Qiranhuang, increased the trait value for KNPR [54]. Wang et al. (2021) identified 10 QTLs for KNPR within the introgression lines (IL) constructed by B73 and K67-11, which could explain 2.23–10.60% of the phenotypic variation of KNPR [55]. Wang et al. (2023) constructed two F_7_RIL populations using TML418 and CML312 as female parents and Ye107 as the male parent. QTL mapping was performed on the two RIL populations using the CIM method. Four QTLs for KNPR were detected in the RIL population constructed by crossing TML418 and Ye107 in two environments, with the corresponding PVE ranging from 5.00% to 10.30%. Three QTLs for KNPR were detected in the RILs constructed by crossing CML312 and Ye107, with the corresponding PVE ranging from 6.60% to 9.80% [56].

### 3.5. QTL for Four Kernel Traits

Liu et al. (2014) conducted a QTL localization analysis of F_2:3_ families derived from Mc and V671 using the CIM method. Single-environment QTL mapping detected a total of six QTLs for KL on chromosomes 2 and 9, explaining 1.18% to 12.92% of the phenotypic variance for KL. Sixteen QTLs for KW were detected on chromosomes other than 7, 8, and 10, explaining 1.70% to 20.51% of the phenotypic variation. Eighteen QTLs for KT were detected on the remaining seven chromosomes, except 3, 6, and 7, explaining 0.84% to 17.98% of the phenotypic variance. Additionally, 15 QTLs for HKW were identified on chromosomes 1, 2, 4, 5, and 7, explaining 0.46% to 12.80% of the phenotypic variance [57]. Raihan et al. (2016) used the CIM method to perform QTL mapping with an F_6_RIL population derived from a cross between Zheng 58 and SK. They detected 18 QTLs for KL, 26 QTLs for KW, 23 QTLs for KT, and 19 QTLs for HKW, explaining 4.00% to 12.54%, 3.15–23.69%, 4.08–17.93%, and 3.66–17.89% of the phenotypic variation for KL, KW, KT, and HKW, respectively [58]. Lan et al. (2018) utilized the F_7_RIL population derived from 178 and K12 for single-environment QTL mapping of four maize kernel traits using the ICIM method. They detected nine QTLs for KL, twelve QTLs for KW, fifteen QTLs for KT, and fourteen QTLs for HKW, with phenotypic variation explained ranging from 7.57% to 21.67%, 6.58% to 23.49%, 6.30% to 22.92%, and 5.80% to 21.54%, respectively [59]. Liu et al. (2020) applied the CIM method to locate QTLs controlling maize kernel traits using the syn10 DH population constructed by Mo17 and B73. They identified a total of fifteen QTLs for KL, twenty-one QTLs for KW, and nine QTLs for KT, with the PVE values ranging from 3.48% to 10.11%, 3.80% to 8.43%, and 3.38% to 15.04%, and no 100-kernel weight QTL was detected in this study [60]. Li et al. (2019) constructed an F_2_ population using L220 and PH4CV. Through QTL mapping, they successfully identified three QTLs for KL, three QTLs for KW, four QTLs for KT, and two QTLs for HKW. The respective phenotypic contribution rates for these QTLs ranged from 5.05% to 7.44%, 6.35% to 13.77%, 4.47% to 7.99%, and 9.47% to 10.86% [61]. Liu et al. (2020) conducted a comprehensive QTL mapping analysis using IF_2_ and RIL populations, both derived from crosses between Ye478 and 08-641. In the IF_2_ population, they identified 14 QTLs for KL, 19 QTLs for KW, 12 QTLs for KT, and 16 QTLs for HKW. The PVE for these QTLs ranged from 0.46% to 16.33%, 3.45% to 8.08%, 3.71% to 9.79%, and 2.76% to 10.32%, respectively. Additionally, in the RIL population, eight QTLs for KL, seven QTLs for KW, two QTLs for KT, and eight QTLs for HKW were identified, with corresponding PVE values of 1.08% to 12.35%, 0.81% to 7.88%, 3.30% to 5.72%, and 4.70% to 8.07%, respectively [62]. Wang (2020) utilized F_2_ and F_2:3_ populations, constructed by SG-5 and SG-7, for the identification of kernel QTLs. Notably, only QTLs for KL and KW were detected. In the F_2_ population, five QTLs for KL (ranging from 4.20% to 14.80%) and seven QTLs for KW (ranging from 4.50% to 23.00%) were identified. Furthermore, in the F_2:3_ population, 15 QTLs for KL (ranging from 4.40% to 15.30%) and 10 QTLs for KW (ranging from 5.00% to 14.50%) were successfully detected [63]. Jiang et al. (2023) employed the CIM method to locate QTLs for kernel traits in an F_2:3_ population constructed from 082 and Ye107. They detected a total of five QTLs for KL, two QTLs for KW, four QTLs for KT, and five QTLs for HKW under single-environment analyses, with the corresponding PVE ranging from 8.30% to 11.56%, 10.20% to 18.02%, 8.90% to 13.19%, and 8.11% to 13.5%, respectively [64].

## 4. Hotspot Bin Regions and Distributional Characteristics of QTLs for Ear-Related Traits on Chromosomes

### 4.1. Hotspot Bin Regions on Chromosomes Associated with Ear-Related Trait QTLs

**Figure 1:**
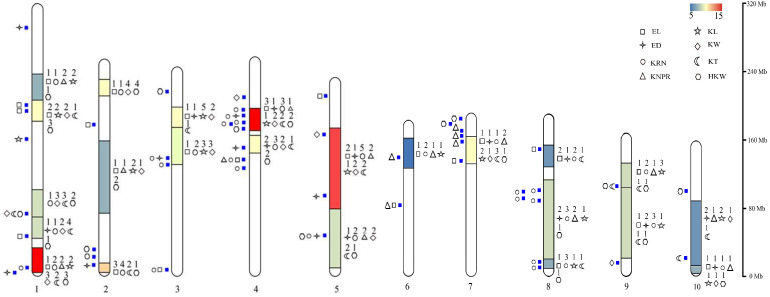
The distribution of QTL hotspots for ear-related traits on maize chromosomes. Note: the frequency of the QTLs mapped for different ear-related traits in the hotspot bin regions are defined in this study (the frequencies range from five to fifteen). The color gradient ranges from blue (indicating fewer QTLs, at least five QTLs) to red (indicating more QTLs, up to fifteen). The different symbols on the right side of the chromosome represent the ear-related traits located in the hotspot bin region. The digital numbers above the symbols represent the number of QTLs for each trait in the bin region. The blue solid square on the left side of the chromosome indicates the physical position of the candidate gene on the chromosome. The symbols on the left side of the blue solid square represent the ear-related traits regulated by the candidate genes.

After statistical analyses, we defined consistent bins that simultaneously localized the relevant QTLs in more than five independent studies as hotspot regions. Using this criterion, we identified a total of 23 hotspot regions, distributed across all 10 chromosomes of maize (Figure 1). Chromosome 1 had the most hotspot bin regions, with five, while chromosome 6 had only one. Most of the hotspot regions were consistent with those found in previous studies, and bin 1.01 and bin 1.02 contained fifteen QTLs for seven traits (excluding ED), bin 4.08 contained fifteen QTLs for eight traits, and bin 6.05 contained at least five QTLs (Table S8). These hotspot regions were consistent with or similar to findings in previous studies [5,57,65,66].

### 4.2. Distributional Characteristics of QTLs for Ear-Related Traits on Maize Chromosomes

Details of the physical locations on the chromosomes, molecular markers, phenotypic variance explained, localization methods, and mapping populations for ear-related QTLs are presented in Supplementary Table S1. The 610 QTLs that have been reported are distributed across all the ten chromosomes, with the highest number of QTLs on chromosome 1 (113), the lowest number on chromosome 6 (27), and the highest number of QTLs associated with KW (123) (Table 1). 

Previous studies have reported an enrichment of ear-related QTLs across all ten maize chromosomes [67]. This review also observed a similar pattern, noting three key features of these enriched QTL regions. (1) QTLs tend to cluster on chromosomes, with an uneven distribution, affecting 2–5 traits each [52,68,69,70], and QTL clusters are chromosome regions that contain multiple QTLs (≥3) related to various traits [71]. (2) QTLs associated with multiple traits can be enriched on the same chromosome, and QTLs related to the same trait can be found on different chromosomes [53]. (3) Major stable QTLs are located within the enriched region [72,73], typically with a PVE greater than 10% [74,75].

**Figure 2:**
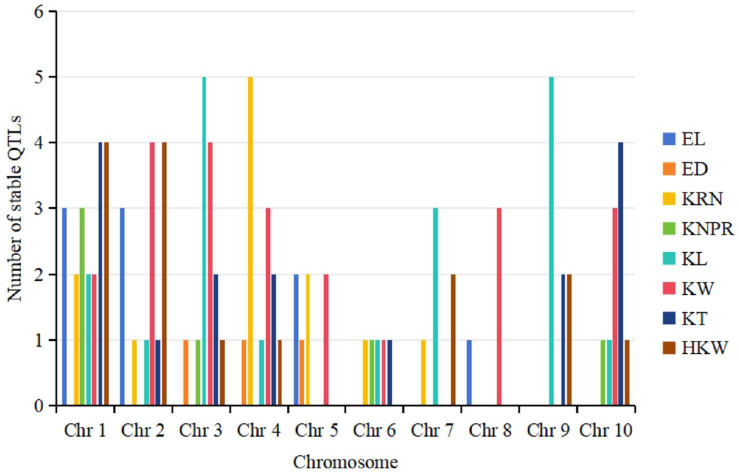
Distribution of stable QTLs for ear-related traits across all ten chromosomes.

Following statistical analysis, a total of 102 stable QTLs associated with ear-related traits were identified (Tables S2 and S3). Stable QTLs are defined as those detected in at least two different environments [76]. Among the 102 stable QTLs, 53 were located in the hotspot bin regions, with the most stable QTLs (6) identified in the hotspot regions, bin 9.03 and bin 9.04 on chromosome 9. The largest number of stable QTLs was observed for KW with 22 QTLs, while ED had the fewest (3) stable QTLs. Furthermore, chromosome 1 exhibited the highest number of stable QTLs (20), encompassing QTLs for seven different traits, except ED. Within this group, KT and HKW had the most stable QTLs (4), while chromosome 8 had only four stable QTLs associated with EL and KW (Figure 2).

**Figure 3:**
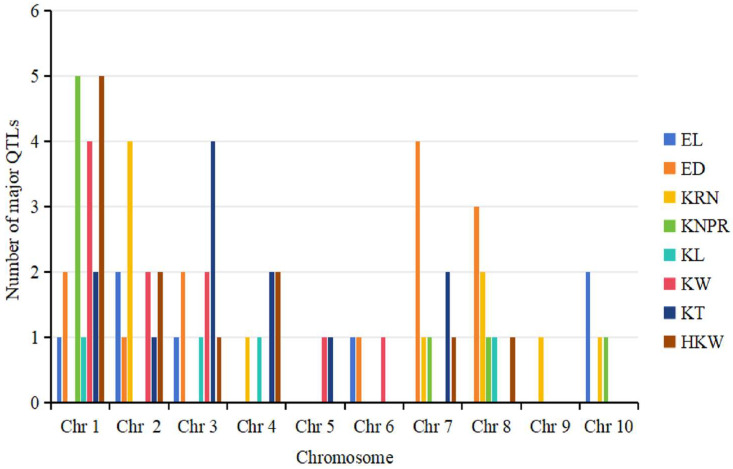
Distribution of major QTLs for ear-related traits across all ten chromosomes.

This study also compiled 76 major QTLs for ear-related traits (Tables S4 and S5), of which 62 were located in the hotspot bin region, and the hotspot region of bin 1.01 and bin 1.02 on chromosome 1 had the highest number of major QTLs (7). Among the traits, ED had the most major QTLs (13), while KL had the fewest (4). Chromosome 1 had the highest number of major QTLs (20), covering seven traits, except KRN. KNPR and HKW had the most major QTLs (5), while chromosome 9 had the fewest, with only one QTL for KRN (Figure 3).

**Figure 4:**
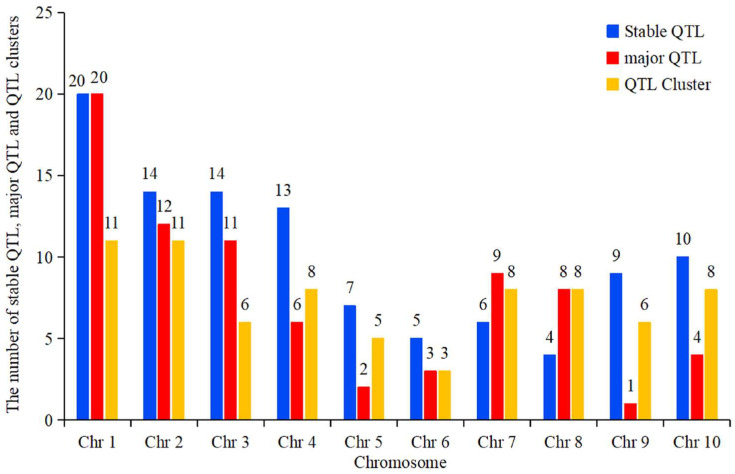
Comparison of the distribution of stable QTLs, major QTLs, and QTL clusters on different chromosomes.

Furthermore, more stable QTLs, major QTLs, and QTL clusters were detected on chromosomes 1 and 2, whereas chromosomes 5, 6, and 9 had fewer of these QTLs (Figure 4). Supplementary Table S6 provides information on QTL clusters for 74 ear-related traits collected. Of these, 62 QTL clusters were located in the hotspot bin regions (Table S7). Among these, most of the QTL clusters were located in bin 1.01 and bin 1.02 (6). The QTL clusters identified were mostly distributed on chromosomes 1 and 2 (11) and were least distributed on chromosomes 5 and 6 (3) (Figure 4). 

## 5. Candidate Genes for Ear-Related Traits

An increasing number of candidate genes for maize ear-related traits are being mapped, but very few have been fine-mapped and validated through cloning. This study compiled a total of 48 candidate genes for maize ear-related traits (Figure 1 and Table 2), of which only six were cloned and validated. These include one EL candidate gene, *Zm00001eb314610* (bin 7.03), in the hotspot region, and five genes in non-hotspot regions: *Zm00001eb123060* (bin 3.02) located near the hotspot region, and *Zm00001eb184050* (bin 4.05), *Zm00001d051012* (bin 4.05), *Zm00001d034629* (bin 1.12), and *Zm00001eb376630* (bin 9.02). Most of the QTLs in the hotspot regions are minor QTLs. However, researchers usually choose major QTLs to clone and verify QTL genes, which may be another reason why there are fewer cloned genes in the hotspots. Another possibility is that the compilation of hotspot bin regions in the study may not be comprehensive enough, and these non-hotspot regions could be considered hotspot regions in other studies or have more QTL found in future studies. The maximum number of candidate genes (8) was located on chromosome 1, with six of them located in the hotspot bin region. The candidate gene *Zm00001eb014970* in bin 1.03 can regulate three traits, viz., KW, KT, and HKW, and the candidate gene *Zm00001d014530* in bin 5.03 can regulate three traits, viz., ED, KRN, and HKW.

In fact, QTL mapping technology and map-based technology have been conducted to identify reliable and stable QTLs for ear-related traits in maize; the following are some examples. One candidate gene controlling KNPR, *Zm00001d038022*, was identified by Brown et al. (2011). This gene encodes a chloroplast pentapeptide repeat-containing protein that is expressed in the spike rachis, affecting EL and possibly positively correlated with KNPR [77]. A gene, *Zm00001eb184050*, associated with KRN and KNPR was validated by Bommert et al. (2013), which codes for a leucine-rich repeat-like receptor protein that shortens EL, thereby affecting KRN and KNPR [78]. In a study by Wang et al. (2019), a major QTL for KRN (KRN1) was cloned and validated. It corresponded to an existing gene (*ids1*/*Ts6*) involved in various biological processes, such as the transition of meristematic tissues from trophic to reproductive stages and the regulation of inflorescence developmental processes [79]. Through fine mapping, the *KRN1* gene was narrowed down to a 6.6 kb genomic fragment, and a gene, *Zm00001d034629*, annotated as a transcription factor of the AP2/EREBP family of transcription factors, was located in close proximity to this 6.6 kb region. A candidate gene, *Zm00001d002737*, affecting ED was identified by Yang et al. (2020) [80]. The gene *Zm00001d016656* was found to affect ED, KRN, and HKW, as identified by Zhang et al. (2022) [20]. A candidate gene, *Zm00001d044081*, was identified and validated from a HKW QTL interval by Sun et al. (2022) [81]. Candidate genes affecting HKW also include *Zm00001eb079220* [82] and *Zm00001eb410780* [83]. Wang et al. (2023) identified one candidate gene, *Zm00001d053080*, associated with KW and KRN, and one potential candidate gene, *Zm00001d011060*, controlling ear length [56].

**Table 2:** Function and physical location of candidate genes for ear-related traits.

Gene (Chromosome)	Predicted Feature	Bin Interval	Physical Interval	Traits Involved	Validation	Reference
Zm00001eb123060 (Chr 3)	RA2 LOB domain protein	3.02	12,830,057–12,832,763	EL, KNPR	Cloned	[84]
Zm00001eb184050 (Chr 4)	Leucine-rich repeat receptor-like protein	4.05	138,680,814–138,683,429	EL, KRN, KNPR	Cloned	[78]
GRMZM5G803935 (Chr 3) *	Encode mir172 microRNA	3.05	144,918,011–144,918,720	ED, KRN	Implication	[38]
Zm00001d027412 (Chr 1) *	Dicer-like 101 (dcl101) protein	1.01–1.02	4,722,956–4,738,332	ED	Implication	[20]
Zm00001d016656 (Chr 5) *	Serine/threonine protein kinase	5.04	171,563,168–171,566,437	ED, KRN, HKW	Implication	
Zm00001d052191 (Chr 4) *	Cupredoxin superfamily protein	4.08	182,743,279–182,744,379	HKW	Implication	
Zm00001d015650 (Chr 5) *	Lycopene β-cyclase andchloroplast-specific lycopene β-cyclase	5.04	103,228,157–103,232,629	ED	Implication	[41]
Zm00001d052442 (Chr 4) *	Auxin effluxcarrier component protein	4.08	190,119,181–190,122,383	ED, KRN	Implication	[85]
Zm00001d034629 (Chr 1)	AP2/EREBP protein	1.12	298,422,859–298,427,050	KRN	Cloned	[79]
Zm00001d038022 (Chr 6) *	Chloroplastic pentatricopeptide repeat-containing protein	6.05	145,415,188–145,419,374	KNPR	Implication	[77]
Zm00001d041584 (Chr 3) *	NB-ARC domain-containing protein	3.05	128,389,890–128,392,834	KRN	Implication	[86]
Zm00001d002737 (Chr 2)	Eukaryotic translation initiation factor 3 subunit C	2.03	20,918,928–20,924,673	ED	Implication	[80]
Zm00001d051328 (Chr 4) *	WRKY transcription factor 12	4.06	154,581,235–154,589,610	ED	Implication	[87]
Zm00001d053080 (Chr 4)	Receptor protein kinase	4.09	212,685,412–212,689,787	KW	Implication	[88]
Zm00001d011060 (Chr 8) *	No annotation	8.05	137,865,777–137,865,788	EL	Implication	
Zm00001d010004 (Chr 8) *	F-box protein At-B	8.03	94,660,952–94,661,221	KRN	Implication	[49]
Zm00001d010007 (Chr 8) *	START domain-containing protein	8.03	94,844,625–94,845,188	KRN	Implication	
Zm00001d010008 (Chr 8) *	Haloacid dehalogenase (HAD)-like hydrolase superfamily protein	8.03	94,945,161–94,946,129	KRN	Implication	
Zm00001d010009 (Chr 8) *	60S ribosomal protein L17	8.03	94,987,331–94,992,881	KRN	Implication	
Zm00001eb199880 (Chr 4) *	SBP-box transcription factor	4.08	205,124,194–205,128,840	KRN	Implication	[89]
Zm00001eb336530 (Chr 8) *	Grass-specific tryptophan aminotransferase	8.02	17,391,163–17,395,311	KRN	Implication	[47]
Zm00001eb336930 (Chr 8) *	Serine/threonine protein kinase	8.02	18,928,567–18,930,699	KRN	Implication	
Zm00001d031906 (Chr 1) *	Dilated protein A24	1.06	206,261,034–206,261,843	EL	Implication	[50]
Zm00001d027721 (Chr 1) *	High-affinity nickel transporter	1.01–1.02	12,140,948–12,146,615	KRN	Implication	
Zm00001eb314610 (Chr 7) *	1-aminocyclopropane-1-carboxylate oxidase2	7.03	129,695,760–129,697,548	EL	Cloned	[22]
Zm00001d022202 (Chr 7)	Protein phosphatase homolog2	7.05	172,755,383–172,761,407	KNPR	Implication	[56]
Zm00001d022168 (Chr 7)	AT hook-containing MAR binding 1-like protein	7.05	171,565,347–171,605,347	KNPR	Implication	
Zm00001d022169 (Chr 7)	RNA polymerase T phage-like 1	7.05	171,565,347–171,605,347	KNPR	Implication	
Zm00001eb019600 (Chr 1) *	GS3-like protein	1.04	71,243,947–71,252,899	KW, KT, HKW	Implication	[57]
Zm00001eb376630 (Chr 9)	RING-type protein with E3 ubiquitin ligase activity	9.02	20,581,735–20,585,861	KW	Cloned	[58]
Zm00001d030895 (Chr 1)	Adenine phosphoribosyltransferase 1 chloroplastic	1.05	166,287,332–166,290,184	KL	Implication	[60]
Zm00001d014530 (Chr 5) *	Phenolic glucoside malonyl transferase	5.03	51,914,095–51,915,783	KW	Implication	
Zm00001d025152 (Chr 10)	Pentatricopeptide repeat-containing protein/PPR	10.04	106,764,011–106,766,200	KT	Implication	
Zm00001d044081 (Chr 3)	Homeobox-leucine zipper protein (ATHB-4)	3.09	218,481,322–218,485,402	HKW	Implication	[81]
Zm00001eb079220 (Chr 2)	Auxin-binding protein	2.04	37,967,776–37,976,021	HKW	Implication	[82]
Zm00001eb410780 (Chr 10) *	Auxin-binding protein homolog4	10.03	27,107,964–27,113,709	HKW	Implication	[83]
Zm00001eb014970 (Chr 1) *	No annotation	1.03	50,584,192–50,589,950	EL	Implication	[90]
Zm00001d046723 (Chr 9) *	EXPANSIN protein family	9.04	103,579,654–103,582,402	KL, HKW	Implication	[91]
GRMZM2G16129 (Chr 2)	7-TM protein	2.06	184,753,214–184,756,735	EL	Implication	[92]
GRMZM2G38381 (Chr 1) *	Protein with an NDR domain	1.06	193,519,623–193,521,732	EL	Implication	
GRMZM2G168371 (Chr 5)	Protein with the Duf640 domain	5.08	214,951,997–214,955,917	EL	Implication	
Zm00001eb331370 (Chr 7)	E3 ubiquitin/ISG15 ligase TRIM25	7.06	174,554,103–174,559,004	HKW	Implication	[40]
Zm00001d022578 (Chr 7)	Ubiquitin-activating enzyme E1 3	7.06	174,785,186–174,790,941	HKW	Implication	
Zm00001d052909 (Chr 4) *	No annotation	4.08	204,448,863–204,453,294	KRN	Implication	[48]
Zm00001d052910 (Chr 4) *	No annotation	4.08	204,476,980–204,480,339	KRN	Implication	
Zm00001d051012 (Chr 4)	Leucine-rich repeat receptor-like protein	4.05	136,764,371–136,769,212	KRN	Cloned	[93]
Zm00001d002641 (Chr 2)	WD40 protein	2.03	17,742,986–17,750,216	KRN	Implication	[23]
Zm00001d036602 (Chr 6)	Serine/threonine protein kinase	6.02	94,190,254–94,199,686	EL, KNPR	Implication	[94]

Note: The candidate gene of marker * indicates that the gene is located in the hotspot bin region.

## 6. Discussion

### 6.1. Consistency of QTLs for Ear-Related Traits

After conducting a comprehensive statistical analysis, we identified a total of 102 stable QTLs related to ear-related traits. These QTLs were mostly distributed across chromosomes 1, 2, 3, 4, and 10, with 53 of them located within hotspot bin regions. Notably, three of these consistent QTLs were co-located by Li et al. (2007) in two different environments, distributed within the bin 1.01 and bin 1.02 region, and associated with four traits: EL, ED, KNPR, and HKW [95]. Additionally, a stable QTL was discovered within bin 5.09 for three traits: EL, KNPR, and HKW. This phenomenon is that one QTL controlling multiple traits can be explained by “pleiotropy”, and these QTLs are often referred to as pleiotropic QTLs [96]. Further, consistent QTLs were also identified in bin 3.08 by Liu et al. (2020) [62], bin 4.07 and bin 4.08 by Lan et al. [59], and bin 8.05 and bin 8.06 by Raihan et al. (2016) [58].

Consistent QTLs play a crucial role in identifying molecular markers tightly linked to various traits and candidate gene selection. However, it is important to note that the majority of QTLs associated with ear-related traits lack consistency. Previous studies have identified two main reasons for this inconsistency: (1) genetic background variability—one significant factor contributing to QTL inconsistency is the genetic background of the mapping population. For instance, Moreno-Gonzalez et al. (1993) found differences in the efficiency of multiple regression estimation for marker-related QTL effects between different generations [97]. Beavis et al. (1994) suggested that genetic background variations were responsible for differences in QTL detection between F_3_ and F_4_ backcross populations, even when the same donor and recurrent parent, such as B73 and Mo17, were used [42]. Li et al. also highlighted that stringent selection during backcross may lead to the elimination of valuable loci from nonrecurrent parents, causing changes in the genetic background and population structure, which, in turn, lead to inconsistencies in detected QTLs [36]. (2) Differential gene action in different environments—another factor contributing to QTL inconsistency is the varying roles of genes in QTLs detected across different environments. Austin observed instances where a QTL was detected in F_2:3_ but not F_6:7_, explaining that the presence of additive (A), dominance (D), or partial dominance (PD) effects in F_2:3_ could lead to the absence of detection in F_6:7_. Notably, among the six QTLs with additive, dominance, or partial dominance effects, the same parental effect was identified in the same genomic region for both F_2:3_ and F_6:7_. When the additive effect is small, QTLs with true over-dominant gene effects in F_2:3_ may go undetected [29]. Liu’s findings further support this notion, especially regarding major QTLs, which displayed additive or partial dominance effects in different environments, underscoring the role of additive and partial dominance effects in the development of maize kernel size and kernel weight [57].

In summary, consistent QTLs controlling ear-related traits offer significant research value by facilitating the integration and enhancement of multiple traits. These QTL regions could be fine-mapped to explore major QTLs and potential candidate genes for the target traits [98]. Consistent QTLs are less influenced by the environment, making them valuable for effectively selecting genes with wide adaptability under varying agro-ecological conditions [57]. The cloning and validation of potential genes associated with consistent QTLs are highly likely to provide valuable insights for molecular breeding, particularly for enhancing drought resistance in maize [72,99,100]. Consequently, the authors believe that subsequent research should focus on the study of major QTLs within these consistent QTL regions and candidate gene exploration [101]. Furthermore, the development of molecular markers from consistent QTLs detected in different populations and environments can significantly expedite the selection and breeding of specific traits in maize [102,103].

### 6.2. The Advantages of Screening Candidate Genes in Hotspot Regions and the Application of Hotspot Regions in Gene Cloning and Maize Breeding in the Future

A comparison with other studies revealed that the candidate gene regions were consistent with the hotspot regions complied in this review. However, it is worth noting that some candidate genes were also found in non-hotspot regions. Zhao et al. (2021) [50] identified an ear length candidate gene *Zm00001d031906* in bin 1.06 of chromosome 1, and Tu et al. (2023) [104] reported five ear length candidate genes, namely *Zm00001d032058*, *Zm00001d032060*, *Zm00001d032062*, *Zm00001d032064*, and *Zm00001d032069,* within the same region. Previous studies have similarly uncovered candidate genes for ear-related traits in hotspot regions, for example, Bortiri et al. (2006) [84], Bommert et al. (2013) [78], Wang et al. (2019) [79], Yang et al. (2020) [80], Liu et al. (2014) [57], Raihan et al. (2016) [58], and Sun et al. (2020) [81], and non-hotspot regions; and non-hotspot regions, for example, Mendes-Moreira et al. (2015) [38], Zhang et al. (2022) [20], Chuck et al. (2014) [89], Forestan et al. (2012) [85], Brown et al. (2011) [77], and Han et al. (2020) [49]. Furthermore, some studies have identified candidate genes for ear-related traits within non-hotspot regions (Liu et al. (2020) [60] and Zhou et al. (2018) [92]).

The authors propose that the 23 identified hotspot bin regions should serve as key areas for researching stable QTLs, major QTLs, and the identification of functional genes associated with ear-related traits. Non-hotspot regions containing major QTLs also hold significant potential for gene discovery research. As research progresses, the number of molecular markers linked to maize ear-related traits is expected to increase, and some of the non-hotspot regions may eventually evolve into hotspot regions.

### 6.3. The Trend of the QTL for Ear-Related Traits in Maize

**Figure 5:**
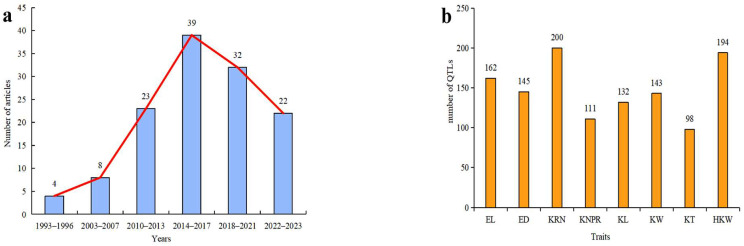
(**a**) Statistical research literature on ear-related traits. (**b**) Statistics of QTLs in the literature on ear-related traits.

The author conducted a search for 128 scientific papers on ear traits in maize spanning from 1993 to 2023 on the journal website. The trend analysis revealed an upward trajectory in the number of research publications from 1993 to 2017, followed by a decline from 2017 to 2023 (Figure 5a). This decline may be attributed to several factors. Firstly, it is plausible that research on QTL mapping of ear-related traits has reached a certain level of maturity, leading to a natural reduction in the number of studies undertaken by scientific researchers. Secondly, the emergence of Genome Structure Variation and SNP-GWAS technology provides researchers with a more sophisticated alternative for analyzing the genetic mechanisms and molecular functions of maize. In addition, a comprehensive review of 39 studies yielded a total of 1184 QTLs associated with eight ear-related traits. Notably, KRN and HKW exhibited the highest frequency of QTLs, while KNPR and KT displayed the lowest. Moving forward, there is a need to intensify research efforts on these latter two traits (Figure 5b).

As science and technology continue to advance, the field of agriculture is steadily moving toward mechanization. Consequently, the demand for specific traits of corn ears is evolving. For corn to be harvested by a machine, corn plants need to be resistant to lodging and ears need to be dried down quickly; thus, maize breeders may set some different goals for ear-related traits. In addition, the maize GY per unit in China is still 40% less than the USA. One important cultivation technology is to increase plant density per unit of land, and this may also change maize breeding goals on ear-related traits; for example, mid-size ears may be preferred compared to big ear sizes in high plant density.

### 6.4. Using of QTLs for Ear-Related Traits in Maize Breeding

The application of maize ear-related QTL traits in molecular marker-assisted selection (MAS) breeding stands as a crucial venue for enhancing breeding efficiency and breeding new varieties. Through the mapping and tagging of QTLs within the maize genome, breeders gain a more precise means of selecting and improving target traits [105]. (1) Molecular marker technology allows researchers to swiftly identify maize traits related to QTLs. These molecular markers, including RFLP, SNP, SSR, etc., enable precise localization of the locus position. This obviates the need for time-consuming field experiments in traditional breeding, thereby enhancing the accuracy of selection. (2) MAS empowers breeders to simultaneously target multiple traits, such as enhancing yield, disease resistance, and adaptability. Through the detection of multiple QTLs, a comprehensive improvement in the performance of maize varieties can be achieved through muti-trait selection. (3) MAS contributes to the acceleration of the breeding process. Traditional breeding often spans numerous years to complete a breeding cycle, but MAS significantly shortens this timeline, facilitating faster market entry for new varieties. (4) Moreover, MAS plays a crucial role in cost reduction in breeding efforts. Identifying plants with the desired trait through genetic markers not only saves time but also diminishes the resources and labor costs associated with cultivation.

In summary, the integration of maize QTLs into molecular marker-assisted selection breeding establishes a scientific foundation for efficient, precise, and swift variety improvement. This approach holds immense value in addressing the escalating demand for food and enhancing stress resistance.

## 7. Future Prospects

In the current stage of research, the study of molecular markers related to ear-related traits faces several significant challenges. (1) The predominant focus on QTL mapping without corresponding efforts in fine mapping to locate causal genes poses a challenge to unraveling the genetic mechanisms underlying ear-related traits. (2) Many studies primarily rely on common mapping populations, such as F_2_, DH, RIL, and BC populations. These populations are often characterized by their modest size and the use of low-density molecular markers, like RFLP and SSR. This practice hinders the precision of QTL mapping. (3) The identification of minor QTLs with low phenotypic variation raises concerns about their practical application value. (4) The insufficient cloning and validation of candidate genes for ear-related traits contribute to the challenges. Moreover, a notable number of research articles report candidate genes without subsequent validation, limiting the reliability of the reported findings. 

In response to the aforementioned challenges, potential solutions include (1) GBS sequencing technology, which presents an opportunity to enhance the precision of QTL localization and narrow down positioning intervals. This method surpasses the limitations of traditional gel-based genotyping assays, allowing for more accurate QTL positioning [7,106]. For instance, in a study on QTL mapping of yield-related traits under drought stress in the reproductive period of rice, Yadav et al. (2019) utilized GBS technology and high-density SNP markers. This strategic use of technology addressed linkage issues between unfavorable and favorable genes often encountered in traditional breeding, leading to efficient and accurate identification of drought resistance genes [107]. (2) Fine mapping and an in-depth exploration of functional genes within stable QTLs and hotspot bin regions identified in prior studies are imperative. A substantial number of candidate genes, stable and major QTLs, and QTL clusters reviewed in this article are located within the hotspot bin region. The strategic investigation of this hotpot bin region is crucial for discovering candidate genes. Therefore, fine mapping and comprehensive studies of the hotspot bin region assume considerable significance in advancing maize breeding efforts. (3) The expansion of mapping population size and the construction of populations with high stability, such as nested association mapping populations and multi-parent advanced generation inter-cross populations, are of paramount importance. Previous research has underscored a strong correlation between the number of mapped QTLs and the size of the mapping population. Smaller mapping populations often yield QTLs with lower accuracy, posing challenges in the detection of minor QTLs [72,108]. For example, in a study by Melchinger et al. (1998), the use of two populations with different sample sizes (N) for QTL localization revealed a notable difference. They identified 107 QTLs when N = 344, whereas only 39 QTLs were detected when N = 107. Notably, only twenty QTLs were consistent between the two populations, underscoring the necessity for a larger sample size to enhance the accuracy of QTL detection [109]. (4) The use of SNP markers is highly recommended for the construction of high-density and high-quality maize genetic maps [39,50]. The density of markers in genetic maps plays a crucial role in the accuracy of QTL localization. High-density SNP markers, in particular, are well suited for fine-mapping QTLs, surpassing other gel-based molecular markers, like SSR and RFLP. Markers closely linked to the target QTLs prove more effective in MAS breeding [110]. (5) Additionally, the integration of previously published genetic maps for ear-related traits and subsequent meta-analysis can significantly enhance mapping accuracy for consistent QTLs. For instance, in a study on maize bursting traits, Kaur et al. (2021) initially identified 99 QTLs. However, QTL meta-analysis reduced this number to 10, facilitating the identification of candidate genes and enhancing the reliability of the targeted QTLs [111]. Similar conclusions were drawn by Van et al. (2017) [112].

## Figures and Tables

**Table 1:** Distribution of QTLs for ear-related traits on all ten chromosomes of maize reported previously.

Trait	Chr 1	Chr 2	Chr 3	Chr 4	Chr 5	Chr6	Chr 7	Chr 8	Chr 9	Chr 10	Total
EL	10	11	6	5	5	5	5	5	4	6	62
ED	11	4	5	5	2	1	5	5	4	5	47
KRN	7	8	3	11	7	3	5	7	7	6	64
KPRN	16	2	4	3	5	2	5	7	2	4	50
KL	14	16	15	4	5	5	10	5	20	4	98
KW	22	18	18	13	8	5	9	13	8	9	123
KT	19	6	14	8	9	2	12	8	4	5	87
HKW	14	10	14	6	6	4	13	3	6	3	79
Total	113	75	79	55	47	27	64	53	55	42	610

## Data Availability

Not applicable.

## Supplementary Materials

The following supporting information can be downloaded at https://www.mdpi.com/article/10.3390/genes15010015/s1, Table S1: Summary information table of QTLs for ear-related traits. Table S2: Distribution of stable QTLs for ear-related traits on chromosomes. Table S3: Summary information table for stable QTLs for ear-related traits. Table S4: Distribution of major QTLs for ear-related traits on chromosomes. Table S5: Summary information table for major QTLs for ear-related traits. Table S6: Summary of QTL clusters for ear-related traits. Table S7: The distribution of stable QTLs, major QTLs, and QTL clusters in 23 hotspot bin regions. Table S8: Distribution of related ear traits in hotspot bin regions.
