# Supplementary material for: Hotspot Regions of Quantitative Trait Loci and Candidate Genes for Ear-Related Traits in Maize: A Literature Review
Source: Genes (Basel). 2023 Dec 21;15(1):15. doi: 10.3390/genes15010015 (PMC10815758; doi:10.3390/genes15010015)
Supplement: Supplementary file 1 [file genes-15-00015-s001.zip › genes-2739067-supplementary.pdf]

Supplementary Table S1. Summary information table of QTLs for ear-related traits.

Combination	Population type	Method	arker type	Trait	Chromosome	Number of QTL	Genetic effect	Gene action	phenotypic variation explained	Reference
Mo17 x H99	$F_{6:7}$	IM	RFLP	EL	1,2,4,6,8	6	A	-	2.10%-5.60%	[29]
48-2 x 5003	$F_{2:3}$	IM	RFLP、SSR	EL	1,2,4,10	4	A、D	A、OD、PD	11.00%-31.50%	[30]
Mo17 x Huangzaosi	$F_{2:3}$	CIM	RFLP、SSR	EL	1,3,4,5,7,9	8	A、D	A、D、OD、PD	2.61%-16.18%	[64]
178 x 9782	RIL	CIM	SSR	EL	4,6,8	4	A	-	5.44%-8.76%	[31]
08-641 x Ye478	RIL	ICIM	SNP	EL	1,3,5,7,8,10	15	A、D	-	3.19%-11.29%	[32]
	IF_2				1,3,5,6,7,8,10	21			2.67%-13.64%	
Yi16 x B73	F_2	ICIM	SSR	EL	4,9	2	A、D	A、PD	6.70%-8.90%	[33]
	$F_{2:3}$				7	1			9.70%	
Mo17 x KW7	RIL	ICIM	SNP	EL	6	1	A	-	18.87%	[34]
Mo17 x H99	$F_{2:3}$	IM	RFLP	ED	1,2,3,6,7,8	6	A、D	D、OD、PD	10.00%-24.00%	[35]
Mo17 x Huangzaosi	F_9	CIM	SSR	ED	4,9	4	A	-	5.68%-8.70%	[37]
PB260 x PB266	$F_{2:3}$	IM	RFLP、SSR	ED	1,3,7	4	A	D、OD、PD	8.70%-19.10%	[38]
Dan32 x N04	BC_2F_2	CIM	SSR	ED	2,4,8,10	6	A	-	4.40%-16.00%	[36]
	F_2				4,5,8,10	3			7.10%-14.80%	
SG-5 x SG-7	F_2	CIM	SNP	ED	1,4,9	5	A、D	-	6.40%-11.60%	[39]
X178 x 9782	$F_{2:3}$	ICIM-ADD	SSR	ED	1,2,3,5,8,10	12	A	-	4.16%-20.44%	[20]
Y32 x Y107	F_7	CIM	SSR	ED	1,9	3	A、D	-	7.10%-10.2%	[41]
SG-5 x SG-7	$F_{2:3}$	CIM	SNP	ED	1,4,10	4	A、D	-	8.50%-12.00%	[40]
[]B73 x Mo17	$F_{2:3}$	IM	RFLP	KRN	4,5,7,9	4	A,D	-	8.00%-10.00%	[42]
Zong3 x 87-1	$F_{2:3}$	MIM	RFL、SSR	KRN	1,2,4,5,6,8,9,10	8	A、D	-	5.80%-13.20%	[43]
L-08-05F x L-14-4B	$F_{2:3}$	CIM	SSR	KRN	1,2,3,7,8,9,10	10	A、D	-	2.40%-16.90%	[44]

B73 x SICAU1212	F_2	CIM	SSR	KRN	2,4,8	7	A、D	A、D、OD、PD	6.78%-36.76%	[45]
Ye478 x Qi319	RIL	CIM	SNP	KRN	2,3,4,5,6,8,9,10	10	A	A	5.39%-7.79%	[47]
V54 x Lian87	$F_{2:3}$	CIM	SSR	KRN	1,2,3,4,5,7,8,9,10	12	A、D	A、D、OD、PD	1.40%-14.95%	[49]
T32 x 319	$F_{2:3}$	ICIM-ADD	SNP	KRN	1,5,8	4	A、D	OD	4.13%-8.33%	[50]
NIL-1133B x B73	F_2<br>$F_{2:3}$	ICIM-ADD	SNP	KRN	4<br>4	1<br>1	A、D	-	32.6%<br>14.61%	[48]
D276 x D72//A188 x Jiao51	Four-way cross	ICIM	SSR	KRN	1,4,6,7,9,10	7	A、D	-	4.74%-11.24%	[46]
Zong3 x 87-1	$F_{2:3}$	CIM	SSR	KNPR	1,2,6,8,9	6	A、D	A、D、OD	5.40%-11.80%	[51]
L26 x 095	F_2	CIM	SSR	KNPR	1,9,10	3	A、D	A、D	17.01%-29.65%	[52]
TY6 x Mo17<br>TY6 x W138"	$F_{2:3}$	CIM	SSR	KNPR	1,3<br>1,3,5,7	3<br>7	A、D	A、OD、PD	3.40%-17.60%<br>0.40%-17.70%	[53]
Baicibaogu x Qiranhuang	$F_{2:3}$	IM	SSR	KNPR	1,4,5,7,	5	A	-	5.40%-11.80%	[54]
TML418 x Ye107<br>CML312 x Ye107	F_7	CIM	SNP	KNPR<br>KNPR	7,8,10<br>1	4<br>3	A	-	5.00%-14.10%<br>6.60%-9.80%	[56]
B73 x K67-1	IL	ICIM	SNP	KNPR	1,4,6,7,8,10	10	A	-	2.23%-10.60%	[55]
Mo17x TY16<br>W138 x TY16	$F_{2:3}$	ICIM	SSR	KNPR	1,3,10<br>1,2,3,5,7	3<br>6	A、D	OD、PD	3.40%-17.160%<br>0.40%-17.80%	[3]
Mc x V671	$F_{2:3}$	CIM	SSR	KL<br>KW<br>KT<br>HKW	2,9<br>1,2,3,4,5,6,9<br>1,2,4,5,8,9,10<br>1,2,4,5,7	6<br>16<br>18<br>15	A、D	A、D、OD、PD	1.18%-12.92%<br>1.70%-20.51%<br>0.84%-17.98%<br>0.46%-12.8%	[57
Zheng58 x SK	F_6	CIM	SSR	KL<br>KW<br>KT	1,2,3,4,5,6,7,8,9<br>1,3,4,5,6,7,8,9,10<br>1,2,3,4,5,7,8,9,10	18<br>26<br>23	A、D	A	4.0%-12.54%<br>3.15%-23.69%<br>4.08%-17.93%	[58]

				HKW	1,2,3,5,6,7,8,9	19			3.66%-17.89%	
178 x K12	F_7	ICIM	SSR	KL	1,2,3,4,8,9	9	A	-	7.57%-21.67%	[59]
				KW	1,3,4,5,7	12			6.58%-23.49	
				KT	1,2,3,4,5,7,8,9	15			6.30%-22.92%	
				HKW	1,2,3,4,7,8,10	14			5.80%-21.54%	
Mo17 x B73	DH	CIM	SNP	KL	1,2,3,5,6,7,9	15	A		3.48%-10.11%	[60]
				KW	1,2,3,4,7,8,9,10	21			3.80%-8.43%	
				KT	1,3,4,6,7,8,10	9			3.38%-15.04%	
082 x Ye107	$F_{2:3}$	CIM	SSR	KL	1,3,7,10	5	A、D	A、D、OD、PD	8.30%-11.56%	[64]
				KW	5,6	2		PD	10.20%-18.02%	
				KT	1,3,7	4		D、OD、PD	8.90%-13.19%	
				HKW	6,7,9,10	5			8.11%-13.56%	
Ye478 x 08-641	IF_2	ICIM-ADD	SNP	KL	1,2,3,5,7,9,10	14	A、D	-	0.46%-16.33%	[62]
				KW	1,2,3,4,5,6,7,8,9,10	19			3.45%-8.08%	
				KT	1,2,3,5,7,8,9	12			3.71%-9.79%	
				HKW	1,3,4,5,7,9,10	16			2.76%-10.32%	
	RIL			KL	1,2,6,9	8			1.08%-12.35%	
				KW	1,2,3,4,9	7			0.81%-7.88%	
				KT	3,7	2			3.30%-5.72%	
				HKW	3,5,6,7,9	8			4.70%-8.07%	
L220 x PH4CV	F_2	ICIM	SNP	KL	2,8,9	3	A、D	-	5.05%-7.44%	[61]
				KW	3,4,8	3			6.35%-13.77%	
				KT	1,2,6	4			4.47%-7.99%	
				HKW	4,9	2			9.47%-10.86%	
SG-5 x SG-7	F_2	CIM	SNP	KL	2,3,9,10	5	A、D	-	4.20%-14.80%	[63]
				KW	1,2,3,8,10	7			4.50%-23.00%	

$F_{2:3}$	KL	2,3,5,6,7,8,9	15	4.40%-15.30%
	KW	1,2,3,8	10	5.00%-14.50%

Supplementary Table S2. Distribution of stable QTLs for ear-related traits on chromosomes.

	Chr 1	Chr 2	Chr 3	Chr 4	Chr 5	Chr 6	Chr 7	Chr 8	Chr 9	Chr 10	Total
EL	3	3	0	0	2	0	0	1	0	0	9
ED	0	0	1	1	1	0	0	0	0	0	3
KRN	2	1	0	5	2	1	1	0	0	0	12
KNPR	3	0	1	0	0	1	0	0	0	1	6
KL	2	1	5	1	0	1	3	0	5	1	19
KW	2	4	4	3	2	1	0	3	0	3	22
KT	4	1	2	2	0	1	0	0	2	4	16
HKW	4	4	1	1	0	0	2	0	2	1	15
Total	20	14	14	13	7	5	6	4	9	10	102

Supplementary Table S3. Summary information table for stable QTLs for ear related traits.

Chromosome	Bin region	Traits involved	Reference
Chr 4	4.09	KRN(2 environments)	[33]
Chr 7	7.00	HKW(2 environments)	
Chr 1	1.05-1.06	EL(2 environments)	[92]
Chr 2	2.06	EL(2 environments)	
Chr 5	5.06-5.08	EL(2 environments)	
Chr 3	3.05	ED(2 environments)	[20]
Chr 2	2.03	EL(3 environments)	
Chr 8	8.08	EL(2 environments)	
Chr 5	5.01	EL(2 environments)	
Chr 1	1.05	HKW(2 environments)	
Chr 4	4.09	KRN(2 environments)	
Chr 9	9.03-9.04	KL(2 environments)	[54]
Chr 8	8.06-8.08	KW(3 environments)	
Chr 10	10.03-10.04	KT(2 environments)	
Chr 9	9.03-9.04	HKW(2 environments)	
Chr 5	5.04	KRN(2 environments)	
Chr 10	10.03-10.04	KNPR(2 environments)	
Chr 7	7.02	KRN(2 environments)	[72]
Chr 2	2.08-2.09	KRN(4 environments)	[49]
Chr 4	4.02-4.05	KRN(2 environments)	
Chr 4	4.06-4.11	KRN(4 environments)	
Chr 1	1.11	KRN(2 environments)	[80]
Chr 1	1.12	KRN(2 environments)	
Chr 1	1.08	KNPR(2 environments)	
Chr 6	6.05	KNPR(2 environments)	
Chr 1	1.09	EL(2 environments)	[53]
Chr 1	1.10	EL(2 environments)	
Chr 2	2.04	EL(2 environments)	
Chr 1	1.04	KNPR(2 environments)	
Chr 1	1.09	KNPR(2 environments)	
Chr 3	3.04	KNPR(2 environments)	
Chr 4	4.07-4.08	KRN(2 environments)	
Chr 5	5.03	KRN(2 environments)	
Chr 6	6.01	KRN(2 environments)	
Chr 4	4.08	ED(2 environments)	
Chr 5	5.03	ED(2 environments)	
Chr 3	3.01	HKW(2 environments)	
Chr 7	7.02	HKW(2 environments)	
Chr 2	2.00-2.02	KT(2 environments)	[70]

Chr 7	4.09	KL(2 environments)	
Chr 2	2.06	HKW(2 environments)	
Chr 4	4.05-4.06	HKW(2 environments)	
Chr 10	10.07	HKW(3 environments)	
Chr 1	1.09-1.10	KL(2 environments)	
Chr 4	4.03-4.05	KL(2 environments)	[99]
Chr 10	10.07	KL(2 environments)	
Chr 3	3.09	KW(3 environments)	
Chr 10	10.07	KW(6 environments)	
Chr 3	3.01-3.03	KT(2 environments)	
Chr 10	10.04	KT(3 environments)	
Chr 9	9.02-9.03	KL(4 environments)	
Chr 9	9.04	KL(4 environments)	
Chr 1	1.02-1.03	KW(5 environments)	
Chr 2	2.05	KW(2 environments)	
Chr 2	2.07	KW(2 environments)	
Chr 2	2.07	KW(3 environments)	
Chr 2	2.08	KW(2 environments)	
Chr 3	3.08	KW(2 environments)	
Chr 4	4.08	KW(3 environments)	
Chr 4	4.08	KW(3 environments)	
Chr 1	1.02	KT(2 environments)	
Chr 1	1.03	KT(2 environments)	
Chr 1	1.04	KT(4 environments)	[57]
Chr 1	1.04-1.05	KT(5 environments)	
Chr 4	4.08	KT(3 environments)	
Chr 9	9.03	KT(3 environments)	
Chr 9	9.04-9.05	KT(2 environments)	
Chr 10	10.02	KT(2 environments)	
Chr 10	10.03	KT(3 environments)	
Chr 1	1.01	HKW(2 environments)	
Chr 1	1.03	HKW(2 environments)	
Chr 1	1.04	HKW(2 environments)	
Chr 2	2.02	HKW(2 environments)	
Chr 2	2.07	HKW(2 environments)	
Chr 2	2.08	HKW(2 environments)	
Chr 1	1.08-1.09	KL(2 environments)	
Chr 2	2.05	KL(2 environments)	
Chr 3	3.05	KL(3 environments)	[60]
Chr 3	3.06	KL(2 environments)	
Chr 6	6.06	KL(2 environments)	

Chr 1	1.08	KW(3 environments)	
Chr 3	3.01	KW(2 environments)	
Chr 4	4.04	KW(2 environments)	
Chr 10	10.05	KW(4 environments)	
Chr 10	10.06	KW(2 environments)	
Chr 4	4.02	KT(4 environments)	
Chr 6	6.06	KT(2 environments)	
Chr 3	3.06-3.08	KL(3 environments)	[64]
Chr 3	3.02-3.03	KL(2 environments)	
Chr 5	5.00	KW(3 environments)	
Chr 6	6.00	KW(3 environments)	
Chr 3	3.06	KT(3 environments)	
Chr 9	9.03-9.04	HKW(2 environments)	
Chr 3	3.04	KW(3 environments)	[63]
Chr 8	8.06	KW(2 environments)	
Chr 8	8.06	KW(2 environments)	
Chr 7	7.03	KL(2 environments)	
Chr 9	9.05	KL(3 environments)	
Chr 9	9.04	KL(2 environments)	
Chr 3	3.04/3.05	KL(2 environments)	[46]
Chr 5	5.03/5.04	KW(2 environments)	
Chr 7	7.02/7.03	KL(2 environments)	

Supplementary Table S4. Distribution of major QTLs for ear-related traits on chromosomes.

	Chr 1	Chr 2	Chr 3	Chr 4	Chr 5	Chr 6	Chr 7	Chr 8	Chr 9	Chr 10	Total
EL	1	2	1	0	0	1	0	0	0	2	7
ED	2	1	2	0	0	1	4	3	0	0	13
KRN	0	4	0	1	0	0	1	2	1	1	10
KNPR	5	0	0	0	0	0	1	1	0	1	8
KL	1	0	1	1	0	0	0	1	0	0	4
KW	4	2	2	0	1	1	0	0	0	0	10
KT	2	1	4	2	1	0	2	0	0	0	12
HKW	5	2	1	2	0	0	1	1	0	0	12
Total	20	12	11	6	2	3	9	8	1	4	76

Supplementary Table S5. Summary information table for major QTLs for ear related traits.

Chromosome	Bin region	Physical interval/bp	Traits	PVE	Reference
Chr 1	1.06	195218120-195221530	EL	12.7%	[30]
Chr 2	2.01	1547887-1552664	EL	31.5%	
Chr 10	10.02	5006086-5010500	EL	11.0%	
Chr 10	10.04	88410123-93648318	EL	11.29%	[32]
Chr 3	3.06	175599768-176836115	EL	13.64%	
Chr 6	6.00	3575200-3577200	EL	18.87%	[34]
Chr 1	1.08	230072700-230078262	ED	24.0%	[35]
Chr 2	3.06	168409685-191113733	ED	21.0%	
Chr 6	6.06	153956114-161325348	ED	19.0%	
Chr 7	7.03	128175453-156050470	ED	16.0%	
Chr 8	8.06	146426603-165267974	ED	13.0%	
Chr 3		-	ED	14.2-18.4%	[38]
Chr 7	-	-	ED	11.2-16.4%	
Chr 7		-	ED	10.9-13.6	
Chr 8	8.03	91588208-99909909	ED	16.0%	[36]
Chr 8	8.03	25014690-70809767	ED	11.4%	
Chr 7	7.03	153701000-155251000	ED	11.6%	[39]
Chr 1	1.03	34257652-36165900	ED	11.71%	[20]
Chr 3	3.05	158552133-158938325	ED	14.13-15.68%	
Chr 9	9.03-9.04	45381581-101748531	KRN	13.2%	[43]
Chr 2	2.02	18,629,118- 19,244,753	KRN	10.6%	[44]
Chr 10	10.02	120,849,364- 121,772,419	KRN	16.9%	
Chr 2	2.02	4895674-15258245	KRN	16.12%	[45]
Chr 2	2.01-2.02	3067260-15258245	KRN	17.07%	
Chr 4	4.06-4.08	222615724-237427552	KRN	12.06%	
Chr 8	8.02-8.03	18612393-33373788	KRN	22.24%	

Chr 8	8.02	16918707-18615853	KRN	36.76%	
Chr 2	2.08-2.09	218356370-225232401	KRN	10.75%-14.75%	[49]
Chr 1	1.01-1.07	6404117-208483075	KNPR	10.4-%-11.8%	[51]
Chr 10	10.02-10.03	4996876-24622715	KNPR	17.01%	[52]
Chr 1	1.09	259363954-259365311	KNPR	14.30%	
Chr 1	1.08	241037980-241285679	KNPR	17.60%	[53]
Chr 1	1.10	278617734-278674963	KNPR	17.70-%	
Chr 1	1.01-1.02	2035-661-23566071	KNPR	11.8%	[54]
Chr 7	7.05	170843056-171585347	KNPR	10.3%	[56]
Chr 8	8.05-8.06	110450438-136744298	KNPR	14.1%	
Chr 1	1.02	14850152-26697162	KW	16.06%	
Chr 2	2.01-2.02	3067260-4706609	KW	10.76%	
Chr 2	2.06-2.07	182622042-193713974	KW	10.78%-11.86%	
Chr 1	1.02-1.03	15805373-33130048	KT	10.79%-12.0%	
Chr 1	1.02-1.03	26684809-43762703	HKW	10.03%-12.23%	
Chr 1	1.04	64243765-82199916	HKW	12.02%-12.20%	
Chr 1	1.05-1.06	170491369-187434251	HKW	12.80%	[57]
Chr 1	1.06-1.07	194693263-206509065	HKW	10.21%	
Chr 3	3.04-3.05	122531243-137558544	KW	10.80%-13.30%	
Chr 1	1.01	52250200-66032549	KT	10.84%	
Chr 3	3.06	188955718-188966168	KT	17.93%	
Chr 1	1.03-1.04	51316436-64403353	HKW	16.04%	
Chr 1	1.03	29264281-7854954	KL	10.3%	
Chr 4	4.10-4.11	238860480-239988267	KL	11.89%	
Chr 8	8.02-8.03	20674613-99909909	KL	12.85%	[58]
Chr 1	1.02-1.03	28926395-34901006	KW	12.12%	
Chr 1	1.04-1.06	55080810-196623386	KW	11.62%	
Chr 1	1.04-1.06	70775896-196623386	KW	12.36%	

Chr 3	3.01-3.02	3478844-5988309	KW	10.16%KT-23.49%	
Chr 2	2.02-2.03	15139189-28680358	KT	10.41%-15.48%	
Chr 3	3.02-3.03	5983469-10080041	KT	20.80%	
Chr 3	3.03	10076687-10457618	KT	18.66%	
Chr 4	4.00-4.01	609416-1080800	KT	10.63%	
Chr 4	4.00	240779448-241788226	KT	22.92%	
Chr 5	4.11	163980240-175656519	KT	12.33%	
Chr 7	7.04	158972709-162851038	KT	13.39%	
Chr 2	2.05	84121511-84122601	HKW	10.77%	
Chr 2	2.08-2.09	218505544-225662933	HKW	13.25%	
Chr 3	3.03	10076687-10457618	HKW	21.54%	
Chr 4	4.06-4.07	161530374-172794795	HKW	17.28%	
Chr 4	4.07-4.08	178275089-184816591	HKW	10.09%	
Chr 8	8.02-8.03	20674613-99909909	HKW	10.73%	
Chr 3	3.06	170265321-179169287	KL	11.36%-11.56%	[64]
Chr 5	5.00	755345-2319068	KW	10.20%-11.96%	
Chr 6	6.00	655718-5447584	KW	14.25%-18.02%	
Chr 3	3.06	179848015-185367968	KT	10.03%-13.19%	
Chr 7	7.03	134114219-152222515	KT	11.93%	
Chr 7	7.00-7.02	2630972-21162260	HKW	13.56%	
Chr 2	2.01	32000000-33000000	EL	25.6%	[40]
Chr 7	7.02	85030593-85036292	KRN	11.24%	[46]

Supplementary Table S6. Summary of QTL clusters for ear-related traits.

Chromosome	Bin region	Traits involved	Reference
Chr 3	3.06	ED、KL	[29]
Chr 8	8.08	ED、KL	
Chr 6	6.05	ED、KRN	
Chr 10	10.06	EL、ED、KL、KRN	
Chr 3	3.05	EL、ED	[30]
Chr 2	2.09	ED、KL	
Chr 3	3.05	ED、KRNP	
Chr 9	9.03	ED、KRN、KL	
Chr 6	6.05	KL、HKW	
Chr 9	9.04	EL、ED	[68]
Chr 4	4.05-4.06	EL、ED	[36]
Chr 8	8.03	ED、HKW	
Chr 8	8.03	ED、KRN、HKW	
Chr 7	7.04-7.05	EL、KNPR	
Chr 7	7.02-7.03	KNR、HKW	
Chr 10	10.02-10.3	ED、KRN	
Chr 10	10.03-10.07	ED、KRN	
Chr 2	2.07-2.08	ED、KRN、KNPR	[31]
Chr 4	4.08	EL、ERN、HKW	
Chr 4	4.09	ERN、KNPR、HKW	
Chr 1	1.02-1.03	KW、KT、HKW	[54]
Chr 2	2.02-2.03	KRN、KL、KT、HKW	

Chr 4	4.05	KL、KW、KT	
Chr 7	7.03	KL、KW、HKW	
Chr 8	8.06-8.09	KL、KW、HKW	
Chr 9	9.03-9.04	KNPR、KL、HKW	
Chr 10	10.03-10.04	KNPR、KL、KT	
Chr 2	2.08	ED、KRN	[80]
Chr 1	1.01	ED、KRN	
Chr 4	4.08	KNR、HKW	
Chr 8	8.02	ED、KRN、KL	[45]
Chr 3	3.07	EL、KRN	[44]
Chr 8	8.03	ED、KRN	
Chr 10	10.03	ED、KRN	
Chr 2	2.05	EL、HKW	[52]
Chr 10	10.03-10.05	ED、KRN、KW	
Chr 10	10.02-10.03	EL、ED、KRN、KNPR、KW	
Chr 9	9.05-9.07	KNPR、KW	
Chr 1	1.08-1.10	EL、KNPR	[53]
Chr 4	4.06-4.09	ED、KRN	
Chr 5	5.06-5.07	KRN、HKW	
Chr 1	1.08-1.10	EL、KNPR	
Chr 4	4.07-4.08	ED、KRN	
Chr 2	bin 2.02	EL、ED、KRN	[32]
Chr 3	bin 3.08	EL、ED、KRN、KNPR	
Chr 6	6.01	KRN、HKW	[70]
Chr 1	1.01	KNPR、HKW	
Chr 5	5.03	KNPR、HKW	
Chr 7	7.04	KNPR、HKW	
Chr 2	2.00-2.02	KT、HKW	[57]
Chr 10	10.04	KT、HKW	

Chr 1	1.02-1.03	KW、KT、HKW	
Chr 1	1.04-1.06	KT、HKW	
Chr 2	2.01-2.02	KW、HKW	
Chr 2	2.05-2.07	KL、KW、HKW	
Chr 2	2.08	KW、HKW	
Chr 4	4.07-4.08	KW、KT、HKW	
Chr 5	5.03	KW、KT、HKW	
Chr 9	9.03-9.04	KL、KT	
Chr 1	1.02-1.03	KW、HKW	
Chr 1	1.08-1.10	KL、KT	
Chr 3	3.05-3.06	KL、KT、HKW	
Chr 7	7.02	KT、HKW	[58]
Chr 7	7.03-7.04	KL、KT、HKW	
Chr 8	8.05-8.07	KL、KW、KT、HKW	
Chr 9	9.07	KL、KW、HKW	
Chr 1	1.02	KL、KW	
Chr 1	1.07	KL、KW	
Chr 2	2.05	KL、KW、HKW	
Chr 5	5.00-5.03	KW、HKW	
Chr 5	5.04	KL、KW	[53]
Chr 7	7.04-7.05	KW、HKW	
Chr 7	7.01	KT、HKW	
Chr 8	8.06	KT、HKW	

Supplementary Table S7. The distribution of stable QTLs, major QTLs and QTL clusters in 23 bin hotspots.

Bin region	Stable QTL/ Individual	Major QTL/ Individual	QTL CLusters/ ndividual
bin 1.01- bin 1.02	3	7	6
bin 1.03	3	6	3
bin 1.04	4	4	1
bin 1.06	1	5	1
bin 1.07	0	2	1
bin 2.02	2	5	4
bin 2.05	2	1	3
bin 2.08	3	2	3
bin 3.05	3	2	3
bin 3.06	3	5	2
bin 4.06	2	2	3
bin 4.08	5	2	4
bin 5.03	2	0	3
bin 5.04	2	0	1
bin 6.05	1	0	2
bin 7.03	2	3	3
bin 8.02	0	4	1
bin 8.03	0	5	4
bin 8.05	0	1	1
bin 9.03	5	1	3
bin 9.04	6	1	3
bin 10.02	1	3	2
bin 10.03	3	1	5
Total	53	62	62

Supplementary Table S8. Distribution table of ear-related traits in hotspot bin regions.

	EL	ED	KRN	KNPR	KL	KW	KT	HKW	Tatal
bin 1.01- bin 1.02*	1	0	2	2	2	3	2	3	15
bin 1.03*	0	1	1	0	0	2	4	1	9
bin 1.04*	0	0	1	0	0	3	3	2	9
bin 1.06*	2	0	0	0	2	2	1	3	10
bin 1.07	1	0	1	2	2	0	0	1	7
bin 2.02	3	0	4	0	0	0	2	1	10
bin 2.05	1	0	0	1	2	1	0	2	7
bin 2.08	1	0	1	0	0	4	0	4	10
bin 3.05*	1	0	2	0	3	3	0	0	9
bin 3.06	1	1	0	0	5	2	1	0	10
bin 4.06*	0	2	3	0	0	2	1	2	10
bin 4.08*	3	1	3	1	1	2	2	2	15
bin 5.03*	0	1	2	2	0	1	2	1	9
bin 5.04*	2	1	5	2	1	2	2	0	15
bin 6.05*	1	0	2	1	1	0	0	0	5
bin 7.03*	1	1	1	2	2	1	3	1	12
bin 8.02*	1	0	3	0	1	0	1	1	7
bin 8.03*	0	2	3	2	1	0	0	1	9
bin 8.05	2	1	2	0	0	0	1	0	6
bin 9.03	1	2	3	0	1	0	1	1	9
bin 9.04*	1	0	2	1	5	0	1	1	11
bin 10.02	1	1	1	1	1	0	1	1	7
bin 10.03*	0	2	1	1	1	0	1	0	6

Note: Candidate genes for ear-related traits are present in the bin region marked with*.
